# Effect of nanoporous TiO_2 _coating and anodized Ca^2+ ^modification of titanium surfaces on early microbial biofilm formation

**DOI:** 10.1186/1472-6831-11-8

**Published:** 2011-03-08

**Authors:** Victoria Fröjd, Paula Linderbäck, Ann Wennerberg, Luis Chávez de Paz, Gunnel Svensäter, Julia R Davies

**Affiliations:** 1 Department of Prosthodontics, Malmo University, Malmo, Sweden; 2 Department of Oral Biology, Malmo University, Malmo, Sweden; 3 Department of Biomaterials, Institute of Clinical Sciences, Sahlgrenska Academy at the University of Gothenburg, Gothenburg, Sweden; 4 Laboratory of Applied Physics, Department of Physics, Chemistry and Biology, Linkoping University, Linkoping, Sweden

## Abstract

**Background:**

The soft tissue around dental implants forms a barrier between the oral environment and the peri-implant bone and a crucial factor for long-term success of therapy is development of a good abutment/soft-tissue seal. Sol-gel derived nanoporous TiO_2 _coatings have been shown to enhance soft-tissue attachment but their effect on adhesion and biofilm formation by oral bacteria is unknown.

**Methods:**

We have investigated how the properties of surfaces that may be used on abutments: turned titanium, sol-gel nanoporous TiO_2 _coated surfaces and anodized Ca^2+ ^modified surfaces, affect biofilm formation by two early colonizers of the oral cavity: *Streptococcus sanguinis *and *Actinomyces naeslundii*. The bacteria were detected using 16S rRNA fluorescence *in situ *hybridization together with confocal laser scanning microscopy.

**Results:**

Interferometry and atomic force microscopy revealed all the surfaces to be smooth (S_a _≤ 0.22 μm). Incubation with a consortium of *S. sanguinis *and *A. naeslundii *showed no differences in adhesion between the surfaces over 2 hours. After 14 hours, the level of biofilm growth was low and again, no differences between the surfaces were seen. The presence of saliva increased the biofilm biovolume of *S. sanguinis *and *A. naeslundii *ten-fold compared to when saliva was absent and this was due to increased adhesion rather than biofilm growth.

**Conclusions:**

Nano-topographical modification of smooth titanium surfaces had no effect on adhesion or early biofilm formation by *S. sanguinis *and *A. naeslundii *as compared to turned surfaces or those treated with anodic oxidation in the presence of Ca^2+^. The presence of saliva led to a significantly greater biofilm biovolume but no significant differences were seen between the test surfaces. These data thus suggest that modification with sol-gel derived nanoporous TiO_2, _which has been shown to improve osseointegration and soft-tissue healing *in vivo*, does not cause greater biofilm formation by the two oral commensal species tested than the other surfaces.

## Background

Titanium dental implants are commonly used to replace lost teeth and much work has been focused on the optimization of the physico-chemical and mechanical properties of implant materials to improve their integration with host bone and soft-tissues. The soft tissue barrier around dental implants serves as a protective seal between the oral environment and the underlying peri-implant bone and one factor proposed to be of importance for the long-term therapeutic success of implant therapy is the development of a good abutment/soft-tissue seal [1]. Various surface modifications of implants, including micro-topographical and chemical surface alterations, have been investigated for their effects on tissue healing and recently, interest has turned to modifications on the nanometer level of resolution [2]. Nanofeatured surfaces are regarded as those with structures smaller than 100 nm in at least one dimension, and nanofeatures have been characterized on at least four commercially available implants [3]. Sol-gel derived nanoporous TiO_2 _coatings have been shown to enhance soft-tissue attachment in rat and dog models [4-6] and an experimental study in human indicated that a significantly greater proportion of oral mucosa was in contact with a nanoporous TiO_2 _surface than with an unmodified surfaces [7].

Surfaces in the oral cavity are rapidly covered with a pellicle of proteins and glycoproteins derived from saliva and gingival crevicular fluid as well as secreted microbial products [8]. The composition, as well as the configuration and density of the proteins in the pellicle, is largely dependent on the physical and chemical nature of the underlying surface and thus the properties of the surface influence bacterial adhesion though the pellicle. Numerous microorganisms in the planktonic phase will be transported to the surface but it is the properties of the conditioning film together with adherence properties of bacteria that determine which organisms attach and initiate biofilm formation. Biofilm formation on tooth surfaces is initiated by adhesion of early colonizers, such as *S. sanguinis *and *A. naeslundii *[9]. The initial colonizers promote the adhesion of secondary colonizers by co-aggregation and microbial interactions leading to maturation of the biofilm [10]. The microbiota of healthy implants is thought to be similar to that seen on tooth surfaces [11,12] and *Streptococcus *spp. and *Actinomyces naeslundii *have been identified as early colonizers on a range of implant material surfaces *in vivo *[13]. As is the case for dental plaque, the capacity for growth and metabolism are the ecological determinants of survival and persistence of oral bacteria on dental implant surfaces. Multiplication and metabolism of adhering micro-organisms ultimately results in the development of a structurally organized microbial community that is in a state of balance with the host [14]. Oral disease may occur when local environmental factors in the biofilm drive the selection and enrichment of putative pathogens belonging to the resident microbiota thus initiating an inflammatory response inducing progressive bone resorption at dental implants [15].

Roughened titanium abutment surfaces have been shown to increase plaque formation *in vivo *[16]. However in comparison, smooth abutment surfaces with surface roughness values of < 0.3 μ m do not promote biofilm formation *in vivo *to the same extent [17]. Smooth, turned (TU) titanium, nanoporous TiO_2 _coated (SG) and anodized Ca^2+ ^modified (OC) surfaces have all been shown to be suitable for osseointegration as well as soft-tissue healing [7,18,19]. In this study, we show that there are no significant differences in early biofilm formation by *S. sanguinis *and *A. naeslundii*, on these three smooth surfaces. However, the presence of saliva led to development of a significantly greater biofilm biovolume by these two colonizers on all surfaces than when saliva was not present.

## Methods

### Preparation of titanium surfaces

Commercially pure turned titanium discs (grade 4), with a diameter of 8 mm and a central hole, were divided into four groups. The original turned discs served as controls (TU) and the other groups were each modified in one of three different ways: sol-gel treatment to create a nanoporous TiO_2 _coat (SG), heat-treated in a similar way to the sol-gel treated discs (HT), or anodically oxidized and calcium treated (OC).

For the sol-gel treatment (SG), discs were cleaned in a basic hydrogen peroxide solution (H_2_O; 30% H_2_O_2_, and 25% NH_4_OH in the ratio 5:1:1) at 85°C for five minutes. After extensive rinsing in distilled water, discs were dried in flowing N_2_. The sol was prepared by mixing solution 1 [10.22 g tetraisopropylorthotitanate, (Merck, Hohenbrunn, Germany) dissolved in 15 ml ethanol] with solution 2 [15 ml of ethanol, 170 μl H_2_O and 840 μl HNO_3_]. After mixing for one hour, 100 μl PEG 400 (Merck, Hohenbrunn, Germany) was added and the solution stirred vigorously. The clear sol was kept at room temperature during aging and the dip-coating process. Dip-coating was performed with using a computer-controlled stepper motor stage with a dipping speed of 30 mm/min, and the discs were sintered in an oven at 500°C (air) for 30 minutes. After heating the discs were ultrasonically cleaned in ethanol for four minutes and finally dried in flowing N_2_. The heat-treated surfaces (HT) which served as controls for the SG surfaces were sintered at 500°C (air) as described above. The anodically oxidized and calcium incorporated (OC) surfaces were prepared by anodic oxidation with an electrolyte consisting of sodium glycerophosphate hydrate (C_3_H_6_(OH)_2_PO_4_Na_2 _× H_2_O) and calcium acetate (Ca(CH_3_COO)_2_) [20,21].

### Characterization of titanium surfaces

To investigate surface roughness on the micrometer level, three discs of each surface were investigated at ten sites using an optical interferometer (MicroXamTM, PhaseShift, Tucson, USA). Each measurement was performed over a 200 × 260 μm area. A high-pass Gaussian filter (50 × 50 μm) was used to separate roughness from errors of form and waviness [22]. The evaluation was performed with the Surfascan software and the images were produced using SPIP™ (Scanning Probe Image Processor, Image Metrology, Denmark). Three different three-dimensional parameters were used to characterize the surface: average height deviation [S_a_(μm)], a spatial parameter - density of summits [S_ds_(1/μm^2^)] and a hybrid parameter including variation in height and spatial direction [S_dr _(%)].

The topography of model silica surfaces, dip-coated as for the SG surfaces, was characterized using atomic force microscopy (AFM 3100, Nanoscope III, Digital Instruments). To characterize the topography on the nanometer level of resolution, the two-dimensional surface parameter, average height deviation [R_a _(nm)], was applied. Thickness of the SG coating was measured with null ellipsometry at λ = 632 nm (Auto-El-III, Rudolph Research, USA). The assumed refractive index of TiO_2 _in anastase crystal structure was n = 2.49.

### Bacterial strains and culture

For biofilm assays the oral type strain *Streptococcus sanguinis *ATCC 10556 and *Actinomyces naeslundii *isolated from dental plaque [23] were used. All strains were routinely maintained on blood agar or in Todd-Hewitt broth (TH) at 37°C in 5% CO_2_.

### Assay for adhesion and early biofilm formation

Immediately prior to bacterial inoculation, discs were cleaned in an ultrasonic bath with Extran MA01^® ^(Merck, Darmstadt, Germany) diluted 1:40 in distilled water, treated with ethanol, and placed in polystyrene 6-well (flat-bottomed) titer plates (MULTIWELL™, Becton Dickinson, Franklin Lakes, NJ, USA). Overnight broth cultures were diluted 1:50 in fresh, pre-warmed Todd Hewitt broth at 37°C in 5% CO_2 _and grown to the mid-exponential growth phase (OD_600 nm_≈0.6). Cultures were then diluted to give final concentrations of approximately 1 × 10^8 ^cells/ml for *S. sanguinis *and 1 × 10^7 ^cells/ml for *A. naeslundii*. 1.5 ml of *S. sanguinis *and 4.5 ml of *A. naeslundii *suspensions were then inoculated into the wells. The microtiter plate was sealed with paraffin tape and incubated at 37°C on a rotary shaker at 300 rpm in 5% CO_2_. Following incubation for 2 and 14 h, the surfaces were rinsed three times with 10 mM potassium phosphate buffer, pH 7.5 (PBS) to remove loosely bound cells. The adherent bacteria were fixed in 4% paraformaldehyde for 16S rRNA hybridization. All biofilm experiments were carried out using independent bacterial cultures three times for each surface type.

For the saliva experiments, unstimulated whole saliva was collected from a healthy volunteer with good oral health, centrifuged for 10 minutes to pellet mucins and bacteria, and the supernatant filter-sterilized (pore size 0.22 μm). Aliquots of bacterial suspensions (1.5 ml of *S. sanguinis *and 4.5 ml of *A. naeslundii*) were centrifuged and the pellet resuspended in 6 ml of the sterile saliva. Bacterial suspensions (containing 10^7 ^colony-forming-units per ml as shown by culturing) were then added to the wells. Plates were shaken gently for 14 hours at 37°C in an atmosphere of 5% CO_2_. After this time, surfaces were rinsed three times with 3 ml PBS, fixed with 4% paraformaldehyde and incubated overnight at 4°C.

### 16S rRNA FISH and confocal laser scanning microscopy

Fixed bacteria on the discs were washed with cold, sterile PBS and subjected to cell membrane permeabilization with 100 μl lysozyme (Sigma, St Louis, MO, USA) [(70 U μl^-1^) in 100 mM Tris-HCl, pH 7.5 (Sigma, St Louis, MO, USA) containing 5 mM EDTA (Merck, Damstadt, Germany)] for 9 minutes at 37°C. After rinsing with ultra-pure water, the bacteria were dehydrated through a series of ethanol washes. Hybridization buffer [0.9 M NaCl, 20 mM Tris-HCl buffer, pH 7.5, with 0.01% sodium dodecyl sulfate (SDS) and 25% formamide] containing 20 ng of labeled oligonucleotide probe ml^-1 ^was pipetted onto the titanium discs. The probe cocktail consisted of the streptococcal probe STR493 (5'-GTTAGCCGTCCCTTTCTGG-3') [24], fluorescently labeled green with ATTO-488 to assess the amount of *S. sanguinis*, and a red-labeled ATTO-565 probe EUB338 (5'-GCTGCCTCCCGTAGGAGT-3') [25] to assess total biofilm volume. Hybridization was carried out at 47°C in a humid chamber for 90 minutes. The surfaces were washed three times with 20 mM Tris-HCl (pH 7.5) containing 5 mM EDTA and 0.01% sodium dodecyl sulfate, and twice with 159 mM NaCl, for 30 and 15 minutes, at 47°C under gentle shaking. Finally, the titanium surfaces were washed with ice-cold ultra-pure water, mounted and glued onto glass slides for analysis using inverted confocal scanning laser microscopy (CSLM) (Eclipse TE2000, Nikon Corporation, Tokyo, Japan). Green fluorescence was provided by an Ar laser (488 nm laser excitation) and red fluorescence was given by a G-HeNe laser (543 nm laser excitation). CLSM images were acquired with an oil immersion objective (×60). Each stack had a substratum coverage field area of 215 × 215 μm, and the z-step was 2 μm. Images were obtained from 15 randomly selected sites per disc.

### Image analysis

**Figure 1:**
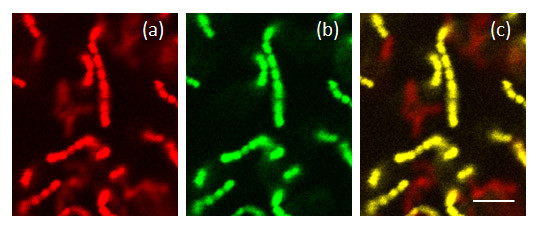
**16S rRNA fluorescence *in situ *hybridization images of *S. sanguinis *and *A. naeslundii *in mono- and dual-species biofilms**. CSLM images showing dual-species biofilms of *S. sanguinis* and *A. naeslundii* (a) stained with the red EUB338 16S rRNA FISH probe, (b) stained with the green STR493 16S rRNA FISH probe or (c) stained with both the probes. The bar shows 4 μm.

The image stacks were converted into TIFF format and analyzed using the bioImage_L software [26] to calculate the structural parameters of the biofilm. *S. sanguinis *takes up both the universal probe EUB338 (red) (Figure 1a) and the streptococcus specific STR493 probe (green) (Figure 1b) and in the images presented these cells appear yellow due to co-localization of the probes (Figure 1c). *A. naeslundii*, which takes up only the EUB338 probe, appears red (Figure 1c). Since the software used could not identify yellow cells, the biovolume of *S. sanguinis *was quantified using the numbers of green cells. The biofilm biovolume of *A. naeslundii *was then calculated indirectly by subtracting the *S. sanguinis *(green) biofilm biovolume from the total.

### Statistical analysis

The non-parametric Mann-Whitney U test was used to analyze differences in biofilm biovolume between the test and control surfaces and p-values < 0.05 were considered significant.

## Results and Discussion

### Characterization of the titanium surfaces

**Figure 2:**
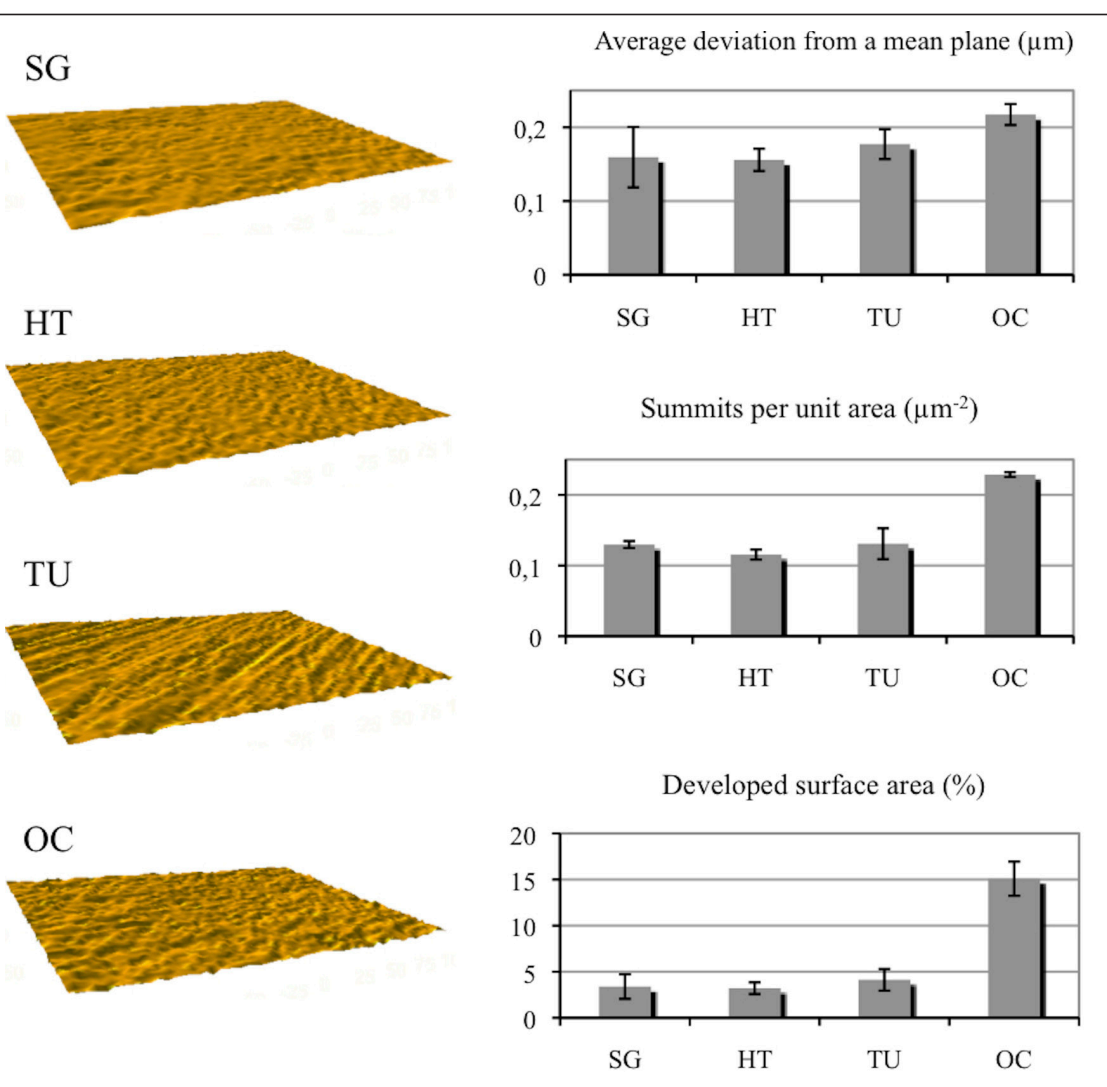
**Interferometry images and surface characteristics of the smooth titanium surfaces**. Images from interferometry were produced with SPIP™. The average height deviation (S_a_), density of summits (S_ds_) and surface enlargement (S_dr_) are shown for the different surfaces; SG (sol-gel derived nanoporous TiO_2 _coated), HT (heat-treated), TU (turned) and OC (anodically oxidized Ca^2+ ^incorporated) surfaces.

Characterization of surface orientation by interferometry revealed that the nanoporous (SG) surface as well as the heat-treated (HT) and anodic oxidized Ca^2+ ^incorporated (OC) surfaces were isotropic while the turned surface (TU) was anisotropic (with oriented surface topography) (Figure 2, left column). Measurements of surface parameters (Figure 2, right column), revealed that the nanoporous (SG) surface had an average surface height deviation (S_a_) of 0.16 ± 0.04 μm while the developed surface area [27] and summit density (S_ds_) were 3 ± 1% and 0.13 ± 0.005 summits/μm^2 ^respectively. This topography was similar to that of the heat-treated control (HT) (S_a _0.16 ± 0.02 μm, S_dr _3 ± 1%, S_ds _0.12 ± 0.007 summits/μm^2^) and the turned (TU) (S_a _0.18 ± 0.02 μm, S_dr _4 ± 1%, S_ds _0.13 ± 0.022 summits/μm^2^) surfaces. The anodic oxidized Ca^2+ ^incorporated (OC) surface however, had higher values for average height deviation (0.22 ± 0.01 μm), developed surface area ratio (15 ± 2%), and summit density (0.23 ± 0.003 summits/μm^2^). Thus, the OC surface had somewhat greater microtopographical structures than the other surfaces investigated and had a greater potential surface area for bacterial interactions. However, despite the differences on the microscale level of roughness between the TU, SG and HT on the one hand and the OC surface on the other, all were categorized as smooth (*i.e*. S_a _< 0.5 μ m) [28].

**Figure 3:**
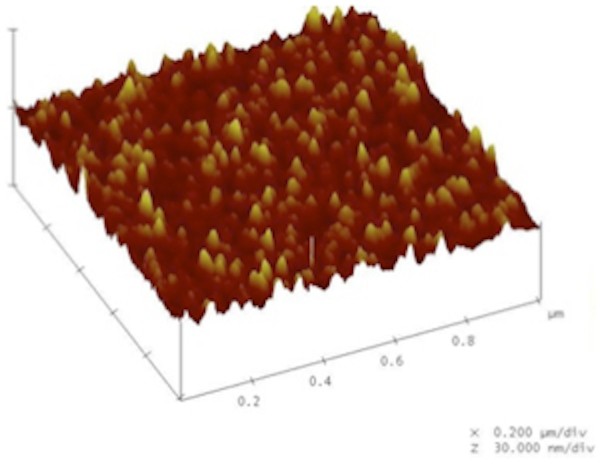
**Characterization of the nanoporous TiO**_**2 **_**coated surface using atomic force microscopy**. A representative AFM image of the nanoporous TiO_2 _coated surface. Note the homogeneous and evenly distributed nanofeatures.

Atomic force microscopy surface imaging of sol-gel derived nanoporous TiO_2 _surfaces showed that the particles were well distributed and organized (Figure 3). The coating led to the formation of nanostructures of a few nanometers up to 100 nm with R_a _1.58 nm. The thickness of the nanoporous TiO_2 _coating, was 90 nm ± 10 nm as measured by ellipsometry.

### Properties of smooth surfaces do not influence adhesion and early biofilm formation by S. sanguinis and A. naeslundii

**Figure 4:**
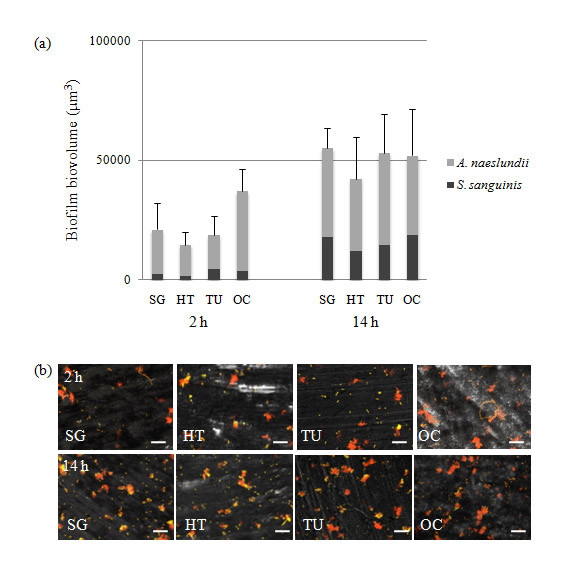
**Biofilm formation by *S. sanguinis *and *A. naeslundii *over 2- and 14-hours on the smooth titanium surfaces**. (a) Graphs showing the mean ± sd of biofilm volume generated from three independent sets of experiments. SG (sol-gel derived nanoporous TiO_2 _coated), HT (heat-treated), TU (turned) and OC (anodically oxidized and Ca^2+ ^incorporated). No significant differences were seen between the surfaces at each time point. (b) Representative images from CSLM of 2- and 14-hour biofilms visualized with 16S rRNA FISH using oligonucleotide probes targeting *S. sanguinis *(STR493 - green) and all bacteria (EUB338 - red). Since both the red EUB338 and the green STR493 probe were taken up by *S. sanguinis*, the cells appear yellow whereas *A. naeslundii *which incorporates only the EUB338 probe appears red. The scale bars represent 10 μm.

The main objective of the present study was to investigate microbial adhesion to nanoporous TiO_2 _(SG) surfaces and compare this to other smooth titanium surfaces used for implant abutments. The model used is compatible with CLSM since the discs can be mounted on glass-slides and viewed directly in the microscope. The amount of bacteria on the surfaces after 2 hours of incubation was considered to represent the level of bacterial adhesion to the surface. After 2 hours, *A. naeslundii *and *S. sanguinis *were present on all surfaces as sparsely distributed cell clusters (Figure 3b - upper panel). The biofilm biovolume on the SG surface was similar to that on the HT control and the TU surface. The OC surface however, showed a slightly higher level of adhesion but this was not-significantly different from that on the other surfaces (p = 0.05) (Figure 4a). Thus it appears that the higher level of microscale roughness seen for the OC surface was not sufficient to protect the bacteria from removal forces. Similar results were obtained in an *in vivo *study where surfaces with a S_a _= 0.21 μm showed somewhat greater levels of adhering microorganisms than those with a S_a _in the range 0.05-0.13 μm [29] and an average roughness in height (R_a_) of 0.2 μm has been proposed as a threshold for significant bacterial adhesion [17]. The proportion of *A. naeslundii *was greater than that of *S. sanguinis *on all surfaces (approximately 85% of the biofilm biovolume) suggesting that, under these conditions, *A. naeslundii *was better able to adhere than *S. sanguinis*.

After 14 hours in the presence of TH growth medium, the adhered bacteria have started to divide and grow and the levels of microbial coverage are thus considered to reflect the initial stages of biofilm formation. However, the levels of growth seen here between two and 14 hours were low and this may reflect the fact that on contact with a surface, planktonic bacteria undergo a transition from exponential growth to a much slower growth rate [30]. No differences in the levels of coverage between the different surfaces could be observed (Figure 4b, lower panel). In accordance with these observations, no differences in the overall biofilm biovolume between the four surfaces were detected. The relative proportion of *S. sanguinis *had increased to 31%, although the levels were still lower than those of *A. naeslundii *(69%). Thus, although the initial levels of adhesion were slightly higher on the OC surface, possibly due to the greater surface area, this was not sustained as the biofilm began to develop.

### Saliva enhances adhesion of S. sanguinis and A. naeslundii in dual-species biofilms

**Figure 5:**
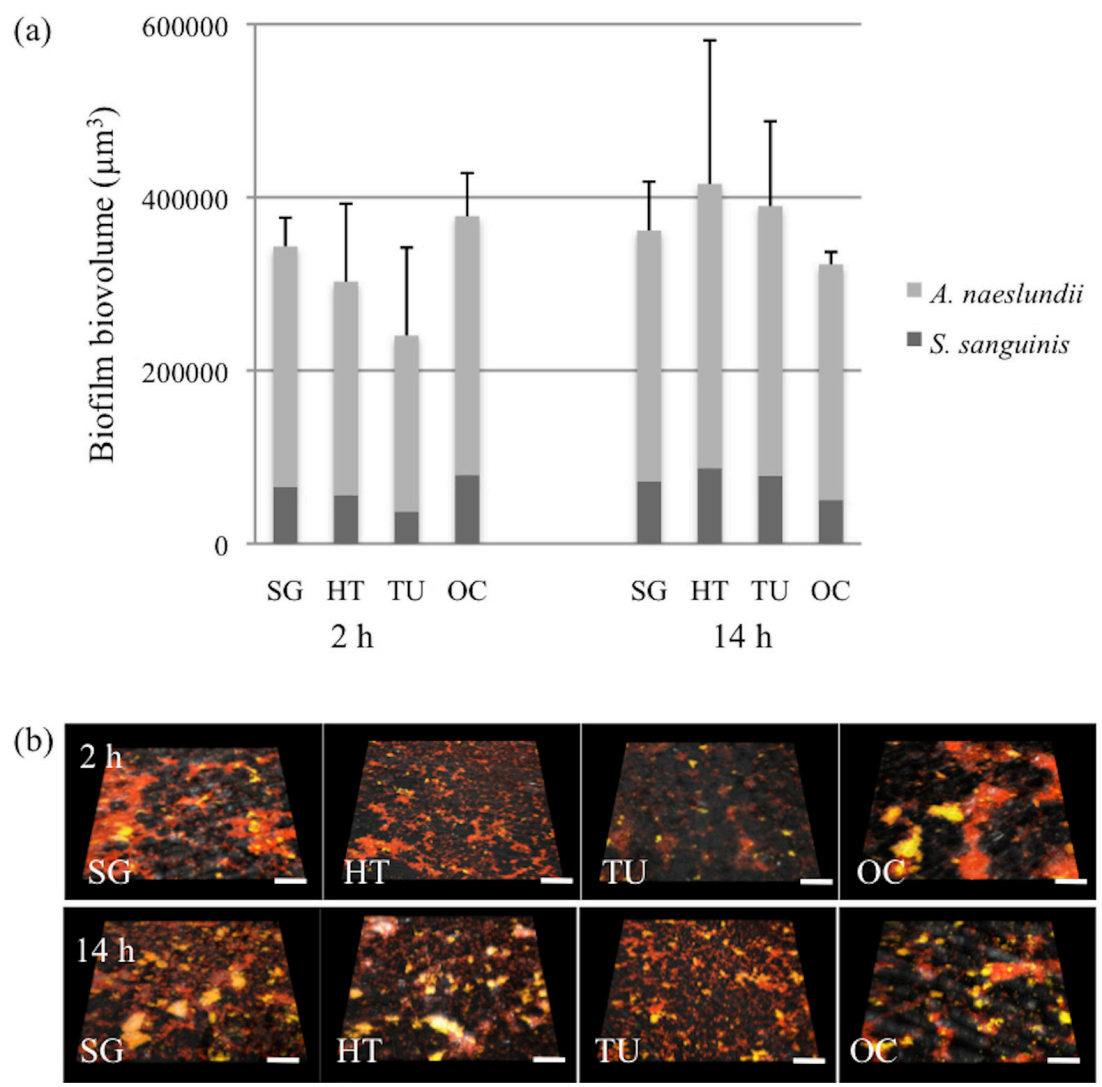
**Biofilm formation by *S. sanguinis *and *A. naeslundii *in the presence of saliva over 2- and 14-hours on the smooth titanium surfaces**. (a) Graphs showing the mean ± sd of biofilm volume generated from three independent sets of experiments. SG (sol-gel derived nanoporous TiO_2 _coated), HT (heat-treated), TU (turned) and OC (anodically oxidized and Ca^2+ ^incorporated). No significant differences were seen between the surfaces at each time point. (b) Representative images from CSLM of two- and 14-hour biofilms visualized with 16S rRNA FISH using oligonucleotide probes targeting *Streptococcus sanguinis *(STR493 - green) and all bacteria (EUB338 - red). Since both the red EUB338 and the green STR493 probe were taken up by *S. sanguinis*, the cells appear yellow whereas *A. naeslundii *which incorporates only the EUB338 probe appears red. The scale bars represent 25 μm.

To investigate if saliva affected surface adhesion, *S. sanguinis *and *A. naeslundii *were suspended in sterile saliva before exposure to the surfaces. This increased the adherence of both species to all surfaces after 2 hours (Figure 5b, upper panel). The bacteria were present as clusters, probably due to aggregation of cells covered with salivary proteins. The increase was up to 11-fold (Figure 5a) as compared to in the absence of saliva (Figure 4a) suggesting that saliva promoted the adhesion of these two species. In contrast, in previous studies pre-coating of titanium surfaces with experimental salivary pellicles was shown not to affect the adherence of *A. naeslundii *[31]. This difference may be attributed to the fact that in the study of Lima *et al*. 2008, bacteria were suspended in nutrient broth rather than saliva.

After 14 hours no major change was seen in the biofilm biovolume indicating that no growth had occurred in the presence of saliva. However, these data are likely to underestimate biofilm formation and growth *in vivo *since in biofilms on dental implant surfaces recruitment of a range of other bacterial species would allow a concerted action to degrade salivary glycoproteins and thus provide nutrients for growth [32]. The different surfaces showed no differences in total biofilm biovolume (p < 0.05) and the proportion of the bacterial species was similar at both 2 and 14 hours, with *A. naeslundii *constituting about 80% of the biovolume.

The model used here enabled the different surfaces to be tested in the presence of saliva. The use of 16S rRNA FISH allows detection of interspecies variations in adhesion and growth as well as the spatial relationships between bacteria on various surfaces. In addition, the extensive rinsing during the 16S rRNA FISH procedure ensures that only truly adhered bacteria are present on the surface during quantification. One drawback of the model is that the gently shaking used here may not accurately reflect the shear forces present at a surface exposed in the oral cavity. However, this could be overcome through the use of a flow-chamber model [33].

## Conclusions

Nano-topographical modification of smooth titanium surfaces did not cause significantly greater adhesion and biofilm formation by *S. sanguinis *and *A. naeslundii in vitro *than was found on turned surfaces or those treated with Ca^2+ ^incorporation during anodic oxidation. In the presence of saliva, adhesion was increased more than ten-fold compared to in the absence of saliva and no differences were seen between the surfaces. These data suggest that modification with sol-gel derived nanoporous TiO_2, _which has been shown to improve soft-tissue healing *in vivo*, does not lead to greater adhesion and initial biofilm formation by the two commensal species tested than the other surfaces. However, it cannot be excluded that over a longer time period in the presence of other bacterial species, greater differences in biofilm formation on the different surfaces may be seen.

## List of abbreviations

CLSM: confocal laser scanning micrscopy; PBS: 10 mM potassium phosphate buffer, pH 7.5; FISH: fluorescence *in situ *hybridization; TU: turned surfaces; OC: anodically oxidized Ca^2+ ^incorporated surfaces; SG: sol-gel derived nanoporous TiO_2 _coated surfaces; HT: heat-treated surfaces.

## Competing interests

The authors declare that they have no competing interests.

## Authors' contributions

VF participated in planning the study, performed most of the laboratory work, and participated in the data analysis and drafting of the manuscript. PL prepared the sol-gel derived surfaces and participated in the drafting of the manuscript. AW participated in the study planning and the drafting of the manuscript. LCP participated in the experimental design and drafting of the manuscript. GS participated in study design, data analysis and drafting of the manuscript. JRD participated in data analysis and drafting of the manuscript. All authors have read and approved the final manuscript.

## Pre-publication history

The pre-publication history for this paper can be accessed here:

http://www.biomedcentral.com/1472-6831/11/8/prepub
